# Supplementary material for: Influenza A Virus Infection Reactivates Human Endogenous Retroviruses Associated with Modulation of Antiviral Immunity
Source: Viruses. 2022 Jul 21;14(7):1591. doi: 10.3390/v14071591 (PMC9320126; doi:10.3390/v14071591)
Supplement: Supplementary file 1 [file viruses-14-01591-s001.zip › MotifAnalysis.zip!/MotifOutput_SC35MdNS1/knownResults.html]

MotifOutput/ - Homer Known Motif Enrichment Results


# Homer Known Motif Enrichment Results (MotifOutput/)

Homer *de novo* Motif Results  
Gene Ontology Enrichment Results  
Known Motif Enrichment Results (txt file)  
Total Target Sequences = 77, Total Background Sequences = 45162

|  |  |  |  |  |  |  |  |  |  |  |  |
| --- | --- | --- | --- | --- | --- | --- | --- | --- | --- | --- | --- |
| Rank | Motif | Name | P-value | log P-pvalue | q-value (Benjamini) | # Target Sequences with Motif | % of Targets Sequences with Motif | # Background Sequences with Motif | % of Background Sequences with Motif | Motif File | SVG |
| 1 | T C G A C T A G A G T C A G T C C G T A C G T A A C G T T A G C T C A G T A C G | NFY(CCAAT)/Promoter/Homer | 1e-30 | -7.113e+01 | 0.0000 | 45.0 | 58.44% | 3229.1 | 7.15% | motif file (matrix) | svg |
| 2 | A T G C G A C T A C G T C T A G A C G T A C G T A C G T C T G A G A T C G C T A A G C T C G T A | Foxa2(Forkhead)/Liver-Foxa2-ChIP-Seq(GSE25694)/Homer | 1e-20 | -4.685e+01 | 0.0000 | 37.0 | 48.05% | 3498.1 | 7.75% | motif file (matrix) | svg |
| 3 | G A C T C G A T C A T G G C T A G C A T C G T A A G T C G C T A G C A T C G A T T A C G G C A T C G T A C A G T G A T C | DMRT6(DM)/Testis-DMRT6-ChIP-Seq(GSE60440)/Homer | 1e-18 | -4.145e+01 | 0.0000 | 22.0 | 28.57% | 989.1 | 2.19% | motif file (matrix) | svg |
| 4 | T C A G C T A G A T G C G A C T T G C A C G T A A G C T A C G T C T G A T A C G | En1(Homeobox)/SUM149-EN1-ChIP-Seq(GSE120957)/Homer | 1e-17 | -4.126e+01 | 0.0000 | 51.0 | 66.23% | 8947.4 | 19.82% | motif file (matrix) | svg |
| 5 | C G T A G C T A C G A T C T A G A C G T G T C A C G T A C G T A A G T C C G T A T G C A T A C G | FoxL2(Forkhead)/Ovary-FoxL2-ChIP-Seq(GSE60858)/Homer | 1e-17 | -4.095e+01 | 0.0000 | 36.0 | 46.75% | 3899.8 | 8.64% | motif file (matrix) | svg |
| 6 | G C A T C G A T C T A G G C T A G C A T C G T A G A T C G C T A C G A T C G A T C T A G C G A T C G T A C G A T G A T C | DMRT1(DM)/Testis-DMRT1-ChIP-Seq(GSE64892)/Homer | 1e-16 | -3.766e+01 | 0.0000 | 22.0 | 28.57% | 1188.8 | 2.63% | motif file (matrix) | svg |
| 7 | G C T A T C G A C G T A C T A G A G C T G T C A G T C A C G T A A G T C C G T A | FOXA1(Forkhead)/MCF7-FOXA1-ChIP-Seq(GSE26831)/Homer | 1e-14 | -3.292e+01 | 0.0000 | 35.0 | 45.45% | 4712.8 | 10.44% | motif file (matrix) | svg |
| 8 | A G C T A G T C A G T C A C G T C T A G A C G T A C G T A C G T C G T A A G T C G A T C C G T A | FOXP1(Forkhead)/H9-FOXP1-ChIP-Seq(GSE31006)/Homer | 1e-13 | -3.212e+01 | 0.0000 | 23.0 | 29.87% | 1753.9 | 3.89% | motif file (matrix) | svg |
| 9 | G C T A T C G A C G T A C T A G A G C T G T C A G T C A C G T A A G T C C G T A | FOXA1(Forkhead)/LNCAP-FOXA1-ChIP-Seq(GSE27824)/Homer | 1e-13 | -3.109e+01 | 0.0000 | 37.0 | 48.05% | 5663.7 | 12.55% | motif file (matrix) | svg |
| 10 | T A C G T A G C G C T A C G A T C T A G A C G T C A G T C A G T G C T A A G T C G T C A G C A T | FOXK2(Forkhead)/U2OS-FOXK2-ChIP-Seq(E-MTAB-2204)/Homer | 1e-11 | -2.646e+01 | 0.0000 | 24.0 | 31.17% | 2551.7 | 5.65% | motif file (matrix) | svg |
| 11 | C G A T C T A G A C G T G T C A C G T A C G T A A G T C C G T A | Foxo3(Forkhead)/U2OS-Foxo3-ChIP-Seq(E-MTAB-2701)/Homer | 1e-10 | -2.405e+01 | 0.0000 | 25.0 | 32.47% | 3136.7 | 6.95% | motif file (matrix) | svg |
| 12 | C T A G T A C G G A T C G T A C G C T A A G C T A G C T G T C A T C G A T A G C | Nanog(Homeobox)/mES-Nanog-ChIP-Seq(GSE11724)/Homer | 1e-8 | -1.892e+01 | 0.0000 | 58.0 | 75.32% | 19266.4 | 42.68% | motif file (matrix) | svg |
| 13 | G C A T G C A T C T G A A C G T C T G A A C G T C G T A C G T A C G T A A G T C G T C A G T C A | Foxf1(Forkhead)/Lung-Foxf1-ChIP-Seq(GSE77951)/Homer | 1e-7 | -1.785e+01 | 0.0000 | 25.0 | 32.47% | 4236.9 | 9.39% | motif file (matrix) | svg |
| 14 | A G C T T G A C C G A T C G A T C T A G A C G T C A G T C A G T G C T A A G T C | FOXK1(Forkhead)/HEK293-FOXK1-ChIP-Seq(GSE51673)/Homer | 1e-7 | -1.727e+01 | 0.0000 | 25.0 | 32.47% | 4362.1 | 9.66% | motif file (matrix) | svg |
| 15 | A T G C C T G A A T C G T A C G A G T C C G A T T C A G C G A T C T A G A G C T G T C A G T C A C G T A A G T C C G T A T A C G C T G A | Fox:Ebox(Forkhead,bHLH)/Panc1-Foxa2-ChIP-Seq(GSE47459)/Homer | 1e-6 | -1.451e+01 | 0.0000 | 23.0 | 29.87% | 4317.5 | 9.56% | motif file (matrix) | svg |
| 16 | T C G A T A G C G T C A A C T G C T A G C G T A C G A T A C T G A C G T A C T G A C T G A C G T | ETS:RUNX(ETS,Runt)/Jurkat-RUNX1-ChIP-Seq(GSE17954)/Homer | 1e-5 | -1.209e+01 | 0.0002 | 7.0 | 9.09% | 396.8 | 0.88% | motif file (matrix) | svg |
| 17 | A G T C A C G T A C T G A G C T A C G T A C G T G T C A A G T C | Foxo1(Forkhead)/RAW-Foxo1-ChIP-Seq(Fan\_et\_al.)/Homer | 1e-5 | -1.189e+01 | 0.0002 | 31.0 | 40.26% | 8323.2 | 18.44% | motif file (matrix) | svg |
| 18 | C A T G A G T C C T G A T G A C A T C G G A C T G T C A A G T C T A G C G A T C | HIF2a(bHLH)/785\_O-HIF2a-ChIP-Seq(GSE34871)/Homer | 1e-3 | -9.160e+00 | 0.0026 | 9.0 | 11.69% | 1094.0 | 2.42% | motif file (matrix) | svg |
| 19 | A T G C A G T C G C T A C G A T C G T A G C A T G C T A C G A T C T A G C A T G T G A C G T C A | CArG(MADS)/PUER-Srf-ChIP-Seq(Sullivan\_et\_al.)/Homer | 1e-2 | -6.607e+00 | 0.0313 | 8.0 | 10.39% | 1253.3 | 2.78% | motif file (matrix) | svg |
| 20 | T A C G C G T A T C A G G A C T C T A G A C T G C A G T T A G C T C G A A C G T G T A C C T A G A G T C A G T C G A T C | ZNF669(Zf)/HEK293-ZNF669.GFP-ChIP-Seq(GSE58341)/Homer | 1e-2 | -5.473e+00 | 0.0924 | 3.0 | 3.90% | 188.1 | 0.42% | motif file (matrix) | svg |
| 21 | A G T C G A T C A G T C C G T A A T C G C A G T A G T C G T A C C T G A A C T G T C A G A G C T A G C T A G C T A G C T | PRDM15(Zf)/ESC-Prdm15-ChIP-Seq(GSE73694)/Homer | 1e-2 | -5.451e+00 | 0.0924 | 14.0 | 18.18% | 3761.9 | 8.33% | motif file (matrix) | svg |
| 22 | A T G C A G T C C T G A A G T C C G A T A C G T A G T C A G T C A C G T A T C G G A C T A C G T | Etv2(ETS)/ES-ER71-ChIP-Seq(GSE59402)/Homer | 1e-2 | -4.793e+00 | 0.1658 | 12.0 | 15.58% | 3224.5 | 7.14% | motif file (matrix) | svg |
